# Age-Related Decrease in Male Extra-Striatal Adenosine A_1_ Receptors Measured **Using** ^11^C-MPDX PET

**DOI:** 10.3389/fphar.2017.00903

**Published:** 2017-12-18

**Authors:** Masahiro Mishina, Yuichi Kimura, Muneyuki Sakata, Kenji Ishii, Keiichi Oda, Jun Toyohara, Kazumi Kimura, Kiichi Ishiwata

**Affiliations:** ^1^Department of Neuro-pathophysiological Imaging, Graduate School of Medicine, Nippon Medical School, Tokyo, Japan; ^2^Research Team for Neuroimaging, Tokyo Metropolitan Institute of Gerontology, Tokyo, Japan; ^3^Department of Neurological Science, Graduate School of Medicine, Nippon Medical School, Tokyo, Japan; ^4^Department of Computational Systems Biology, Faculty of Biology-Oriented Science and Technology, Kindai University, Kinokawa, Japan; ^5^Department of Radiological Technology, Faculty of Health Sciences, Hokkaido University of Science, Sapporo, Japan; ^6^Institute of Cyclotron and Drug Discovery Research, Southern TOHOKU Research Institute for Neuroscience, Koriyama, Japan; ^7^Department of Biofunctional Imaging, Fukushima Medical University, Fukushima, Japan

**Keywords:** adenosine A_1_ receptor, aging, positron emission tomography, humans, cerebral cortex, thalamus

## Abstract

Adenosine A_1_ receptors (A_1_Rs) are widely distributed throughout the entire human brain, while adenosine A_2A_ receptors (A_2A_Rs) are present in dopamine-rich areas of the brain, such as the basal ganglia. A past study using autoradiography reported a reduced binding ability of A_1_R in the striatum of old rats. We developed positron emission tomography (PET) ligands for mapping the adenosine receptors and we successfully visualized the A_1_Rs using 8-dicyclopropylmethyl-1-^11^C-methyl-3-propylxanthine (^11^C-MPDX). We previously reported that the density of A_1_Rs decreased with age in the human striatum, although we could not observe an age-related change in A_2A_Rs. The aim of this study was to investigate the age-related change of the density of A_1_Rs in the thalamus and cerebral cortices of healthy participants using ^11^C-MPDX PET. We recruited eight young (22.0 ± 1.7 years) and nine elderly healthy male volunteers (65.7 ± 8.0 years). A dynamic series of decay-corrected PET scans was performed for 60 min starting with the injection of ^11^C-MPDX. We placed the circular regions of interest of 10 mm in diameter in ^11^C-MPDX PET images. The values for the binding potential (*BP*_ND_) of ^11^C-MPDX in the thalamus, and frontal, temporal, occipital, and parietal cortices were calculated using a graphical analysis, wherein the reference region was the cerebellum. *BP*_ND_ of ^11^C-MPDX was significantly lower in elderly participants than young participants in the thalamus, and frontal, temporal, occipital, and parietal cortices. In the human brain, we could observe the age-related decrease in the distribution of A_1_Rs.

## Introduction

Medical development has increased the average human lifespan (Vaupel, 2010). Cognitive functions such as memory often decline as humans age (van Geldorp et al., 2015), and aging is the major risk factor for Alzheimer’s disease (Fjell et al., 2014a). Human brain becomes atrophied with the aging (Fjell et al., 2014b), although brain atrophy remains mild in some elderly people called “superager” or “successful aging” whose cognitive functions remain intact with age (Depp and Jeste, 2006; Harrison et al., 2012; Sun et al., 2016).

Neuronal systems responsible for brain function are known to decline with age (Morrison and Baxter, 2012). In the human brain, neuroimaging studies revealed that endogenous dopamine, and dopamine transporter, D_1_ and D_2_ receptors and aromatic L-amino acid decarboxylase decrease with age (Suhara et al., 1991; Reeves et al., 2002; Ishibashi et al., 2009), while monoamine oxidase B increases with age (Reeves et al., 2002). Such age-related decrease has also been reported in the cholinergic, glutamatergic and γ-aminobutyric acid (GABA)ergic systems (Segovia et al., 2001; Rissman et al., 2007; Schliebs and Arendt, 2011).

**FIGURE 1:**
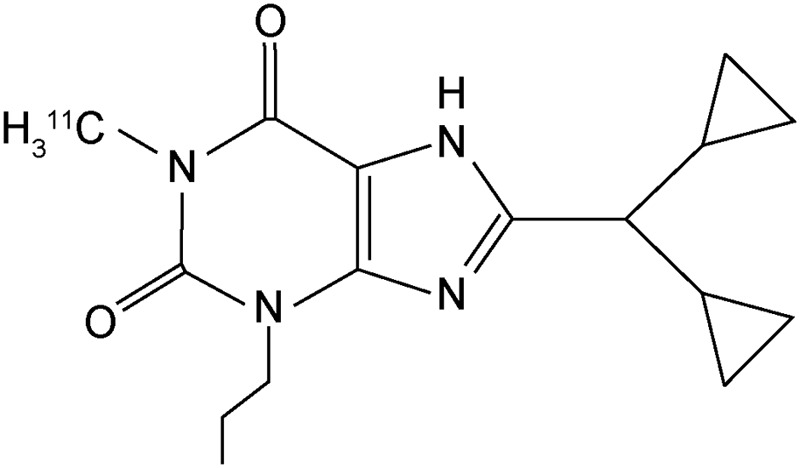
Chemical structures of ^11^C-MPDX.

In the adenosinergic system, animal studies reported that the age-related changes differ in the subtypes: adenosine A_1_ (A_1_R) and A_2A_ receptors (A_2A_R) (Cunha et al., 1995, 2001; Lopes et al., 1999; Rebola et al., 2003; Meerlo et al., 2004). We developed ligands for positron emission tomography (PET) to map the adenosine receptors, and successfully visualized the A_1_Rs using 8-dicyclopropylmethyl-1-^11^C-methyl-3-propylxanthine (^11^C-MPDX, **Figure 1**) (Fukumitsu et al., 2005) and the A_2A_Rs using [7-methyl-^11^C]-(*E*)-8-(3,4,5-trimethoxystyryl)-1,3,7-trimethylxanthine (^11^C-TMSX) (Ishiwata et al., 2000a,b, 2002). Using ^11^C-MPDX and ^11^C-TMSX PET, we previously reported that the density of A_1_Rs decreased with age in the human striatum, although we could not observe an age-related change in A_2A_Rs (Mishina et al., 2012). In order to compare A_1_R and A_2A_R, we did not study the density of A_1_Rs other than striatum in the past paper (Mishina et al., 2012). Because A_2A_Rs are enriched in the striatum (Fredholm and Svenningsson, 2003; Mishina et al., 2007), while A_1_Rs are widely distributed throughout the entire human brain (Fukumitsu et al., 2005). Another human PET study reported that the binding ability of ^18^F-8-cyclopentyl-3-(3-fluoropropyl)-1-propylxanthine (^18^F-CPFPX), an A_1_R ligand, was negatively correlated with age in the cerebral cortices and thalamus in addition to the striatum (Meyer et al., 2007). The A_1_Rs in the cerebral cortex are thought to help regulate the GABAergic and glutamatergic systems (Albasanz et al., 2002; Cunha-Reis et al., 2008; Ferreira et al., 2014), while A_1_Rs in the striatum are mainly responsible for the regulation of the D_1_ receptor in medium spiny neurons (Ferre et al., 1994; Yabuuchi et al., 2006). We hypothesized that the A_1_Rs may decrease with age in the thalamus and cerebral cortices. The aim of this study was to investigate the age-related change in the density of A_1_Rs in the thalamus and cerebral cortices of healthy participants using ^11^C-MPDX PET.

## Materials and Methods

### Participants

We recruited eight young healthy (mean age ± standard deviation [SD], 22.0 ± 1.7 years, age range, 20–25 years) and nine elderly male volunteers (65.7 ± 8.0 years, age range, 51–77 years). The participants were all Japanese and right-handed. None of the participants had a history of neurological diseases or any abnormalities upon physical or neurological examinations. Additionally, none took any medications known to affect the brain function or had a history of alcoholism. They had no medical history of bronchial asthma, and did not regularly use theophylline, a nonselective A_1_R and A_2A_R antagonist.

This study was approved by the Ethics Committee of Tokyo Metropolitan Institute of Gerontology. Written, informed consent was obtained from all participants in this study.

### Magnetic Resonance Imaging (MRI)

MRI was performed in the Tokyo Metropolitan Geriatric Hospital with three-dimensional spoiled gradient-recalled echo (SPGR) imaging and a SIGNA 1.5 Tesla machine (General Electric, Waukesha, WI, United States). The MRI images validated that the participants had no neurological diseases, such as stroke or brain tumors, and were used as a reference for placing the regions of interest (ROIs) on the PET images.

### PET Measurements

Positron emission tomography was performed in the Positron Medical Center, Tokyo Metropolitan Institute of Gerontology with a SET-2400W PET scanner (Shimadzu, Kyoto, Japan). The scanner had an axial field-of-view of 20 cm, acquired 63 slices at a center-to-center interval of 3.125 mm, and had a spatial resolution of 4.4 mm full width at half maximum (FWHM) and a z-axis resolution of 6.5 mm FWHM (Fujiwara et al., 1997). All participants were asked to abstain from caffeinated beverages, such as tea and coffee, and foods containing chocolate, for 12 h prior to undergoing the ^11^C-MPDX PET, because caffeine is a non-selective adenosine receptor antagonist (Statland and Demas, 1980). ^11^C-MPDX was prepared as described previously (Fukumitsu et al., 2005). To obtain an attenuation map to correct for photon attenuation, an 8-min transmission scan with a rotating ^68^Ga/^68^Ge line source was recorded before the radiotracer injection. Starting at the time of injection, a dynamic series of decay-corrected PET scans was performed for 60 min in a two-dimensional scanning mode. The injected dose of ^11^C-MPDX was 639 ± 77 MBq (16.0 ± 11.6 nmol). Specific activity at the time of injection ranged from 14.6 to 129.5 TBq/mmol (59.5 ± 36.8 TBq/mmol). The total number of frames was 27 and the frame arrangements were 6 × 10 s, 3 × 30 s, 5 × 1 min, 5 × 2.5 min, and 8 × 5 min.

### Image Processing

Image analyses were carried out with the medical image processing software Dr. View/Linux R2.5 (AJS, Tokyo, Japan) implemented in CentOS 5.4 (The CentOS Project^1^) and Parallels Desktop 5.0.9344 (Parallels Holdings, Renton, WA, United States).

We generated early images, which were considered similar to images for cerebral blood flow, by summing frames from 0 to 10 min (Mishina et al., 2000). The MRI image was three-dimensionally registered to the early image of each participant. The early images and the registered MRI images were used as references for placing each ROI on the PET images from the dynamic scans. Circular ROIs with 10 mm in diameter were placed bilaterally on the PET images over the thalamus, frontal, temporal, occipital, and parietal cortex. We also placed the circular ROI over the cerebellar hemisphere as a reference region for kinetic analysis. Averaged tissue time activity curves (tTACs) were derived from the dynamic data and ROI, and data were used to calculate the standardized uptake value.

Kinetic analyses of the tTACs were performed using programs implemented on MATLAB version 7.04 (The Mathworks, Natick, MA, United States) and a General Kinetic Modeling Tool in PMOD 3.0 (PMOD Technologies, Zurich, Switzerland). The values for the binding potential (*BP*_ND_) of ^11^C-MPDX (Kimura et al., 2004) in the regions were calculated using an averaged tTAC and a graphical analysis with the cerebellum as the reference region (Logan, 2003), where the *k*_2_ of the reference region was 0.23/min that was the averaged *k*_2_ as presented in the **Table 1** of a past paper (Kimura et al., 2004) and the starting time for the analysis was 10 min after the administration. We confirmed that the *BP*_ND_ of ^11^C-MPDX was suitable for evaluating the distribution of A_1_Rs (Kimura et al., 2004).

### Statistical Analysis

Statistical computations were performed using the software package JMP Pro version 13.2.0 (SAS Institute, Cary, NC, United States). If the variance had a significant difference between young and elderly groups with Bartlett’s test, Welch’s t-test were used to compare the *BP*_ND_ of ^11^C-MPDX. If not, unpaired *t*-tests were used instead. We also used the regression analysis to compare the age with the *BP*_ND_ of participants. The level of significance was set at *p* < 0.05.

## Results

**FIGURE 2:**
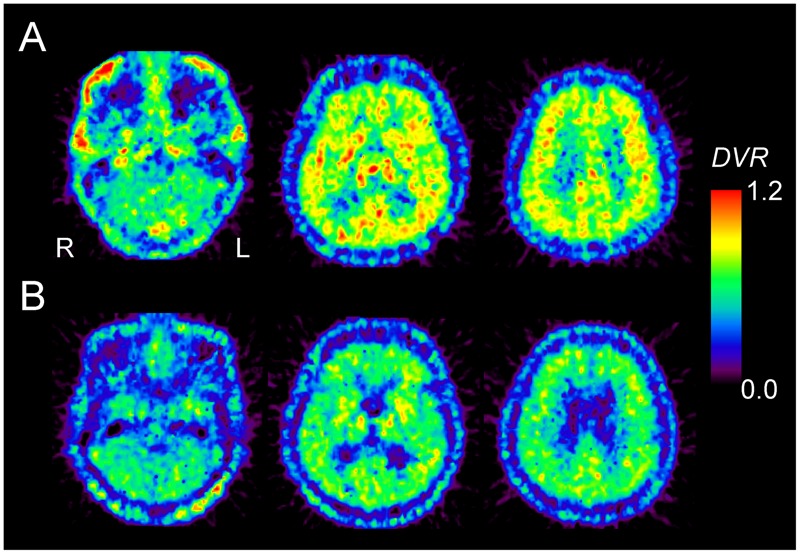
^11^C-MPDX PET images for a 22-year-old male subject **(A)** and a 77-year-old male subject **(B)**. The pixel values for the PET images of ^11^C-MPDX are visualized as the distribution volume ratio (*DVR*), because the brain anatomy is unclear in the *BP*_ND_ images of ^11^C-MPDX. Note that we use the values for the binding potential (*BP*_ND_) in the kinetic analysis for ^11^C-MPDX PET in **Figures 3 and 4**.

**FIGURE 3:**
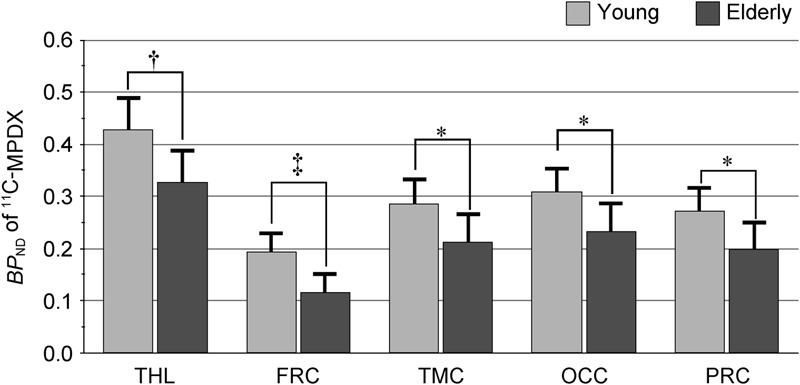
*BP*_ND_ of ^11^C-MPDX in the young and elderly participants. *BP*_ND_ of ^11^C-MPDX was significantly smaller in elderly participants than young participants in the thalamus, frontal, temporal, occipital, and parietal cortex. THL, thalamus; FRC, frontal cortex; TMC, temporal cortex; OCC, occipital cortex; PRC, parietal cortex. ^∗^*p* < 0.05, ^†^*p* < 0.01, ^‡^*p* < 0.001, unpaired *t*-test.

**Figure 2** shows representative ^11^C-MPDX PET images. *BP*_ND_ of ^11^C-MPDX was significantly smaller in elderly participants than young participants in the thalamus (young *vs.* elderly; 0.43 ± 0.07 *vs.* 0.33 ± 0.06, *p* < 0.01; unpaired *t*-test), frontal (0.19 ± 0.04*vs.* 0.12 ± 0.04, *p* < 0.001; unpaired *t*-test), temporal (0.29 ± 0.05 *vs.* 0.21 ± 0.06, *p* < 0.05; unpaired *t*-test), occipital (0.31 ± 0.05 *vs.* 0.23 ± 0.06, *p* < 0.05; unpaired *t*-test), and parietal cortices (0.27 ± 0.05 *vs.* 0.20 ± 0.06, *p* < 0.05; unpaired *t*-test, **Figure 3**).

**FIGURE 4:**
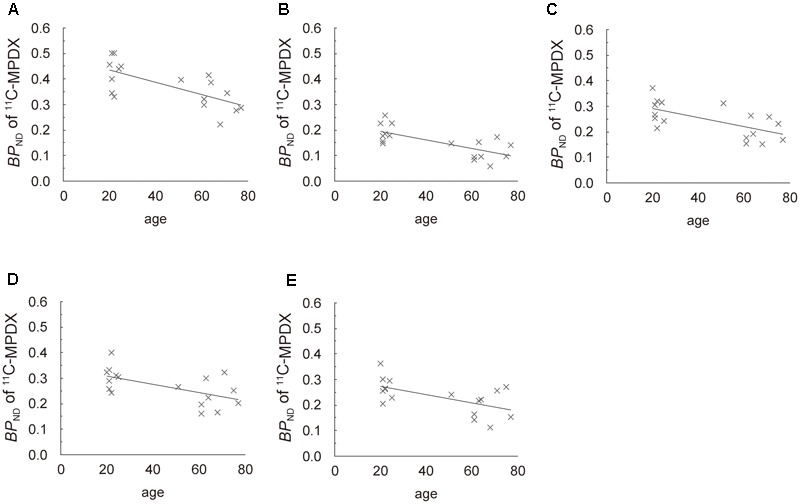
Scattergrams of age *vs. BP*_ND_ of ^11^C-MPDX. *BP*_ND_ of ^11^C-MPDX was negatively correlated with age in the thalamus **(A)**, and frontal **(B)**, temporal **(C)**, occipital **(D)**, and parietal cortices **(E)**.

Regression analyses also showed that the *BP*_ND_ of ^11^C-MPDX was negatively correlated with age in the thalamus (*R*^2^ = 0.458; *p* < 0.005, **Figure 4A**), and frontal (*R*^2^ = 0.500; *p* < 0.005, **Figure 4B**), temporal (*R*^2^ = 0.402; *p* < 0.01, **Figure 4C**), occipital (*R*^2^ = 0.338; *p* < 0.05, **Figure 4D**), and parietal cortices (*R*^2^ = 0.349; *p* < 0.05, **Figure 4E**).

## Discussion

In the human thalamus and cerebral cortices, we observed an age-related decrease of the *BP*_ND_ of ^11^C-MPDX, which is in line with previous findings in the striatum (Mishina et al., 2012). Two reasons are considered to have reduced the *BP*_ND_ of ^11^C-MPDX, namely (1) reduced binding site and (2) increased endogenous adenosine. If the concentration of extracellular adenosine is increased, the estimated apparent *BP*_ND_ using ^11^C-MPDX is decreased by competition at the A_1_R between endogenous adenosine and ^11^C-MPDX. Extracellular adenosine levels in the striatum were not affected by age (Burnstock and Dale, 2015), although no data are available on adenosine in the cerebral cortex. In addition, the affinity of ^11^C-MPDX is higher than that of adenosine (Noguchi et al., 1997; Müller and Jacobson, 2011; Mishina and Ishiwata, 2014). These findings weaken the counter-hypothesis that the age-related decrease in the *BP*_ND_ of ^11^C-MPDX involved an increase in adenosine. Therefore, the results involved an age-related decrease in A_1_Rs in the human brain.

Some studies have revealed differences in age-related changes between A_1_Rs and A_2A_Rs (Burnstock and Dale, 2015). The age-related changes vary in different brain regions. Cunha et al. (1995) studied age-related changes in rats, using [^3^H]2-[4-(2-p-carboxyethyl)phenylamino]-5′-*N*-ethylcarboxamidoadenosine (^3^H-CGS 21680) for A_1_Rs and [^3^H]-1,3-dipropyl-8-cyclopentylxanthine (^3^H-DPCPX) for A_2A_Rs. In their study, A_1_Rs were decreased in the cerebral cortex and hippocampus, but A_2A_Rs were increased only in the cerebral cortex of aged rats. No significant changes were observed in A_1_R of the striatum and in A_2A_R of both the hippocampus and the striatum. An autoradiography study using [^3^H]N6-cyclohexyladenosine demonstrated that there was an age-dependent reduction in A_1_Rs in most of the brain areas of rats, but that the degree of the reduction varied among regions (Meerlo et al., 2004). Efficiency of A_2A_Rs to modulate synaptic transmission in the hippocampus was decreased in aged rats (Sebastiao et al., 2000), although the efficiency of A_2A_Rs was increased by aging (Lopes et al., 1999; Rebola et al., 2003). It seems that there are age-related changes in the balance between inhibitory A_1_R- and excitatory A_2A_R-mediated actions.

A limitation the lack of data on participants’ daily sleep state. A_1_R and A_2A_R play an important role in regulating sleep (Basheer et al., 2000; Scammell et al., 2001; Urade et al., 2003; Elmenhorst et al., 2007; Oishi et al., 2008). Some studies showed that sleep deprivation increased A_1_R (Basheer et al., 2000; Elmenhorst et al., 2007). Another study suggested that endogenous adenosine suppressed the histaminergic system via A_1_R to promote non-rapid eye movement sleep (Oishi et al., 2008). Many elderly people are suffering from insomnia (Dijk et al., 2000; Ohayon et al., 2004; Colrain, 2011). The age-related changes to A_1_R may be associated with insomnia in elderly people.

Another limitation of this study was the lack of data on participants’ daily caffeine intake. In this study, we restricted caffeine consumption in the 12 h prior to performing PET scans, because caffeine is a non-selective adenosine receptor antagonist. Many elderly Japanese people habitually drink green tea after meals (Kuriyama et al., 2006), although the overall caffeine consumption is attributed more to coffee than to tea in Japan (Fredholm et al., 1999). Animal studies reported that chronic administration of caffeine increases the density of adenosine receptors (Green and Stiles, 1986; Nehlig et al., 1992; Li et al., 2008), although human data are sparse. Another limitation the lack of data on participants’ daily sleep state. Adenosine is involved in circadian rhythm and sleep (Bjorness and Greene, 2009), and adenosine inhibits the arousal system via A_1_R and induces sleep (Oishi et al., 2008). Elderly people often have sleep disorders. Further studies are needed to reveal the relationship between chronic caffeine consumption and A_1_R density.

Our study was only comprised of males. A post-mortem study reported adenosine level in the cerebral cortex was higher in male than in female (Kovacs et al., 2010). However, some papers showed that there was no significant gender effect on A_1_Rs in the human brain (Ulas et al., 1993; Glass et al., 1996; Meyer et al., 2007).

## Author Contributions

MM wrote the first draft of the manuscript. MM, KeI, KO, JT, and KiI performed PET examinations. YK and MS performed kinetic analyses. KK and KiI supervised the study. All authors reviewed, commented on, and approved the final report.

## Conflict of Interest Statement

The authors declare that the research was conducted in the absence of any commercial or financial relationships that could be construed as a potential conflict of interest.

## Footnotes

1 http://www.centos.org/
